# Ionic Thermoelectric‐Powered Resistive Sensors

**DOI:** 10.1002/advs.202413093

**Published:** 2024-12-16

**Authors:** Mingna Liao, Hongting Ma, Nan Zhu, Magnus P. Jonsson, Dan Zhao

**Affiliations:** ^1^ Laboratory of Organic Electronics Department of Science and Technology Linköping University Norrköping SE‐601 74 Sweden; ^2^ Wallenberg Wood Science Center Linköping University Norrköping SE‐601 74 Sweden; ^3^ School of Chemistry Dalian University of Technology Dalian Liaoning 116024 China

**Keywords:** ionic thermoelectric supercapacitor, power supplying, resistive sensor

## Abstract

Ionic thermoelectric supercapacitors (ITESCs) are noted for their high ionic Seebeck coefficient (α) to convert thermal energy into electrical current through charging. This work demonstrates the utilization of the charging and discharging current from ITESCs to directly operate resistive sensors. The humidity monitoring is powered by applying a periodic temperature gradient to a connected ITESC. By leveraging these properties and residual environmental heat, ITESCs can offer a promising method for autonomously powered portable sensors.

## Graphical abstract

The operation of a resistive sensor is directly powered by the charging and discharging current generated from an ionic thermoelectric supercapacitor (ITESC) under a temperature difference. The concept presents a promising approach for ITESCs to directly power portable sensors.

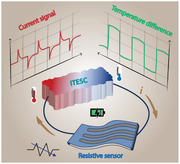

## 1 Introduction

Portable and wearable sensors are innovative devices designed to monitor and collect data on various physiological and environmental parameters in real‐time.^[^ ^1^ ^]^ These sensors play an integral role in healthcare, sports and fitness, and industrial applications by providing valuable insights into an individual's health, activity levels, and environmental exposures.^[^ ^2^ ^]^ Resistive sensors are the most applied ones due to their simple structure, cost‐effectiveness, and ease of maintenance. The fundamental principle of operating a resistive sensor is monitoring the resistance of an internal material that reacts to changes in targeting physical quantities. Translated by a designed circuit, the variations in non‐electrical physical quantities, such as pressure, temperature, humidity, and weight can be read by the change in voltage or current. The ability to reliably convert physical phenomena into electrical signals makes them crucial in automated testing and control systems.^[^ ^3^, ^4^ ^]^

In response to the need for portability and wearability in applications, the design of resistive sensors is shifting toward miniaturization.^[^ ^5^, ^6^, ^7^, ^8^, ^9^ ^]^ The major challenge is ensuring a reliable power supply.^[^ ^10^ ^]^ In recent years, tremendous studies have focused on self‐powered supplying in order to facilitate better construction of integrated sensor systems.^[^ ^11^, ^12^ ^]^ Traditionally, self‐powered sensors referred to devices that harness sufficient energy from the presence of an analyte to support sensor operation and signal transmission, without relying on external power sources such as battery or power outlet.^[^ ^13^ ^]^ Nowadays, the definition of self‐powered sensors has expanded to include those that harvest energy from the environment like solar power,^[^ ^14^ ^]^ vibrations,^[^ ^15^ ^]^ and temperature differentials via pyroelectric and thermoelectric effects.^[^ ^16^, ^17^, ^18^, ^19^ ^]^ The evolution of these technologies has made sensor applications more flexible and sustainable by eliminating the use of batteries.

In recent years, ionic thermoelectrics have been explored intensively as a novel direction of thermoelectrics, motivated by high ionic Seebeck coefficient (α) and sustainable material composition.^[^ ^20^ ^]^ Thermal energy can be harvested by the ionic thermoelectric effect via charging a supercapacitor, which forms the device concept of the ionic thermoelectric supercapacitor (ITESC).^[^ ^21^, ^22^ ^]^ However, the energy density stored in a single ITESC is typically in the range of 1–100 µJ cm^−2[^ ^21^, ^22^, ^23^ ^]^ and the power varies over time, making it complicated to power electronic devices in a conventional manner. To date, there are no powering applications reported using an ITESC. In this work, we instead apply the charging/discharging current generated from ITESCs to directly operate resistive sensors. The resistance change in the sensor can be determined from changes in peak intensity of the charging and discharging current of the ITESC upon a periodically varying temperature difference. We demonstrate the concept by forming an ITESC‐driven sensing system for a resistive humidity sensor. Directly applying the charging/discharging current in an ITESC as the driving power for resistive sensors provides a new path of utilizing the ionic thermoelectric effect. It can harness the residual heat generated in the environment, enhancing the self‐powered capabilities of portable sensors.

## 2 Results and Discussion

### 2.1 Device Concept

**Figure 1:**
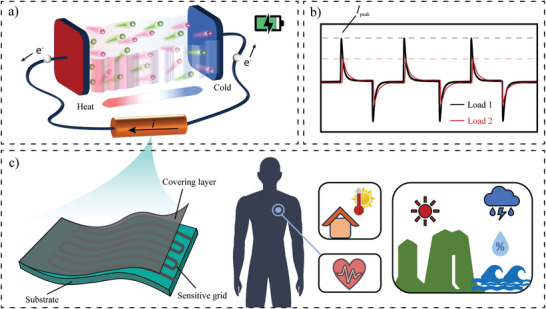
Schematic of the proposed self‐powered sensor system. a) Illustration of an ITESC. b) Illustrative curve of output current signals from an ITESC connected to load resistances. c) Illustration of the structure of a typical resistive sensor and possible applications.

**Figure** **1**a illustrates an ionic thermoelectric supercapacitor (ITESC) under a temperature difference (∆*T*) across the device. The ions in the electrolyte thermodiffuse toward the cold side and accumulate at the interface between the electrolyte and the electrodes. A thermal potential (Δ*V*_thermal_) is established between the two electrodes due to different thermodiffusion rates of the cation and anion.^[^ ^24^, ^25^, ^26^ ^]^ In turn, Δ*V*_thermal_ induces a current flow from one electrode to the other if an external circuit is connected, which resembles the charging of the supercapacitor. Removing Δ*T* and disconnecting the external circuit eliminates the accumulated ions from thermodiffusion while the charge remains at the electrodes. The stored charge in the electrodes can be discharged (consumed) by connecting a load again. If the same load resistance is connected for the whole operation cycle, a pair of symmetric charging and discharging currents can be measured with opposite signs (Figure 1b). The ITESC can experience charging and discharging cycles when applying intermittent Δ*T*.^[^ ^27^ ^]^ As illustrated in Figure 1c, our sensor platform connects a resistive sensor based on a covering layer and sensitive grids to an ITESC as the load resistance. Typically, resistive sensors function by transforming changes in non‐electrical physical parameters such as pressure, temperature, humidity, and weight into corresponding variations in resistance, which can be converted into current or voltage signals through a reading circuit. We present the concept of self‐powered resistive sensor by directly applying the charging/discharging current from an ITESC under Δ*T* to the connected resistive sensor as an external load. The change in resistance in response to physical quantities by resistive sensor can be converted into corresponding changes in the intensity of the peak current (*I*_peak_) (Figure 1b).

### 2.2 ITESC Characterization

**Figure 2:**
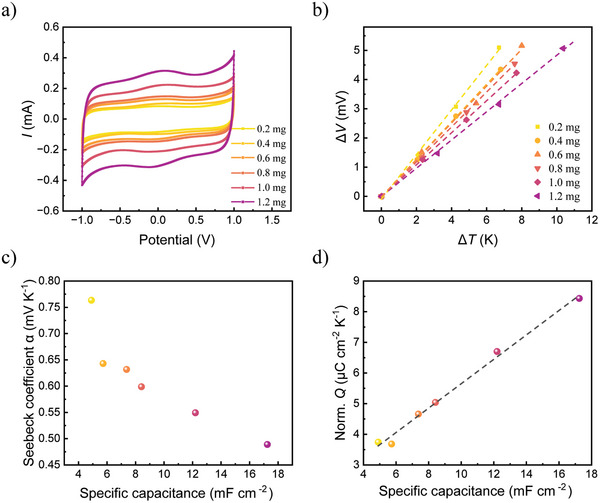
The effect of capacitance. a) CV curves for ITESCs with electrodes loaded with 0.2–1.2 mg of CNTs, respectively. b) Open circuit voltage changes (∆*V*) as a function of ∆*T* across the ITESCs with different CNTs loadings. The dashed lines are linear fit to experimental results in corresponding colors. c) Extracted ionic Seebeck coefficients (α) of ITESCs with different specific capacitances. d) The normalized (by Δ*T*) amount of transferred charge *Q* calculated from *Q*_*norm*._ =  *C*α for ITESCs of different capacitances. The dashed line is a linear fit to the calculation results.

The vertical ITESCs (2 mm thickness, 0.79 cm^2^ effective area) in this study consist of an ionic thermoelectric electrolyte 1‐ethyl‐3‐methylimidazolium ethyl sulfate (EMIM:ES) with 10% hydroxyethyl cellulose (HEC), sandwiched between two Au electrodes loaded with carboxylated carbon nanotubes (CNTs). Cyclic voltammetry (CV) results shown in **Figure** **2**a demonstrate the increasing trend in capacitance (C) with increasing amounts of CNTs. The extracted capacitance values from the CV are 3.9, 4.5, 5.8, 6.7, 9.6, and 13.6 mF for ITESC electrodes loaded with 0.2, 0.4, 0.6, 0.8, 1, and 1.2 mg CNTs, respectively. Impedance measurements (Figure S1, Supporting Information) also confirm that the capacitance of the ITESCs increases with increasing amounts of CNTs. The ionic Seebeck coefficient (α) of ITESCs was determined as the slope from the linear fitting of the change in ∆*V* (the change of voltage difference between the two electrodes) versus ∆*T* (Figure 2b). As shown in Figure 2c, α decreases with the addition of CNTs. This may be due to additional interface effects from residual negatively charged groups on the carboxylated CNTs, which has been observed in previous work.^[^ ^27^ ^]^ Combining the effect of CNT loading on the Seebeck coefficient and capacitance, the normalized (by Δ*T*) amount of transferred charge (Q) (calculated from *Q*  =  *C*Δ*V*  =  *C*αΔ*T*, *Q*_*norm*._ =  *C*α) for different ITESCs still increases with capacitance (as shown in Figure 2d).

### 2.3 The External Current of ITESCs

When charging an ITESC during the establishment of the thermal voltage, the current (*I*) through the external circuit follows:

(1) $$I=\frac{\mathrm{dQ}}{\mathrm{dt}}\approx C\frac{d\Delta V}{\mathrm{dt}}\approx C\alpha \frac{d\Delta T}{\mathrm{dt}}$$

**Figure 3:**
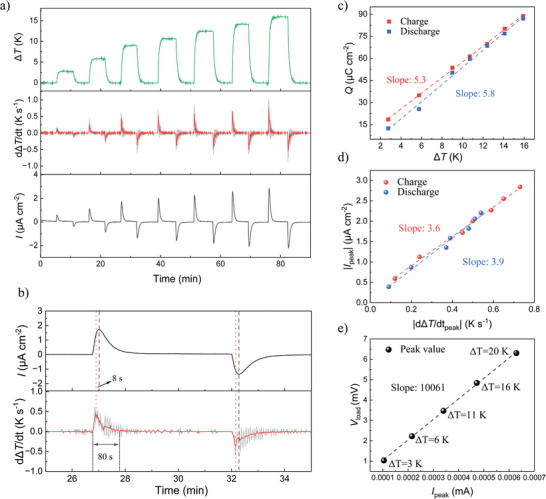
The effect of different applied temperature difference changing rate. a) Applied ∆*T* (top panel), changing rate of ∆*T* (d∆*T*/dt, middle panel), and output current (bottom panel) of an ITESC for different ∆*T*. Raw data and smoothed data of d∆*T*/dt (Savitzky‐Golay filter, points of window 20) are shown as grey and red colors, respectively. b) Enlarged profile of output current and d∆*T*/dt in 26–34 min cycle where ∆*T* = 9 K. c) The amount of transferred charge *Q* of an ITESC of different ∆*T*. d) The *I*_peak_ for different d∆*T*/dt upon charging and discharging process. e) The peak values of *V*_load_ as a function of *I*_peak_ with different ∆*T* and d∆*T*/dt. The black dashed line is a linear fit to the experimental results, with a slope of 10 kΩ.

The first approximate sign assumes that the time needed for charging the supercapacitor is negligible compared to the rate change of Δ*V*. The second approximately sign accounts for the fact that thermodiffusion takes time, which should give a delay between the evolution of Δ*T* and Δ*V*. We first investigated the external current generated from an ITESC with fixed capacitance when applying different ∆*T* and thereby different temperature changing rates. We connected an ITESC with 1 mg CNTs loaded electrodes to an external load of 1 kΩ and recorded the output current under varying ∆*T* conditions. As shown in **Figure** **3**a, the peak magnitude of the changing rate of ∆*T* (marked as d∆*T*/d*t*, middle panel) increases with Δ*T* (top panel). The linear fitting of the correlation upon applying and removing Δ*T* is shown in Figure S2 (Supporting Information). Distinct pairs of charging and discharging current (bottom panel) were observed along with applying and removing the series of ∆*T*. A cycle between 26 and 34 min (where Δ*T* = 9 K) was enlarged in Figure 3b to reveal the details of the peaks. We observe a clear correlation between *I* and d∆*T*/d*t*, with a delay between the time of the peak positions as expected. As the ionic thermodiffusion is the induced by Δ*T*, the change of the thermovoltage follows Δ*T* while will always be slower than the change of Δ*T*. However, this delay is relatively short compared to the total time to establish a stable Δ*T*. The amount of transferred charge *Q* from integrating the current increases linear with Δ*T* following *Q*  =  *C*αΔ*T* (Figure 3c). The amount of *Q* from discharging was slightly less than charging due to a small portion of self‐discharges. The slope of the amount of charge versus Δ*T* is 5.3µC cm^−2^ K^−1^, which is relatively close to the theoretical value of 6.6 µC cm^−2^ K^−1^ (the multiply of the capacitance and Seebeck coefficient). The extracted absolute values of peak current density (|*I*_peak_|) for different 𝑑Δ𝑇/𝑑𝑡 in the charging and discharging process are plotted in Figure 3d. The linear fitting of *I*_peak_ versus 𝑑Δ𝑇/𝑑𝑡 gives a slope of 3.9 µC cm^−2^ K^−1^ that is reduced from the theoretical value of 6.6 µC cm^−2^ K^−1^. This is because of the delay of the current development compared to the change of Δ*T*, which is evidenced in Figure 3b.

The correlation between the *I*_peak_ and 𝑑Δ𝑇/𝑑𝑡 was further confirmed by applying/removing the same ∆*T* (10 K) at different rates (Figure S3, Note S1, Supporting Information). The amount of transferred charge during charging and discharging remains similar regardless of the ∆*T* changing rate (Figure S3b, Supporting Information), while the *I*_peak_ of charging processes increases linearly with the d∆*T*/dt (Figure S3c, Supporting Information). The results imply that obtaining a reproducible external current to reliably determine changes in load resistance will require the application of the same heating rate to the ITESC. One alternative approach, which circumvents the influence of heating conditions, is to measure the output voltage and current synchronously. As a demonstration, we connected an extra resistor *R*_1_ in series to *R*_load_ to record the output current through the external circuit (Figure S4, Supporting Information). Note that the resistive contribution from *R*_1_ (100 Ω) is negligible because it is much smaller than the *R*_load_ (10 kΩ). As shown in Figure 3e, the series peak values of output voltage (*V*_peak_) linearly change with *I*_peak_ for different applied Δ*T* (with different d∆*T*/dt), which give a reproducible read *R*_load_. This result confirms that the impact of different heating conditions can be effectively removed from the sensing operation.

### 2.4 The Impact of Capacitance

**Figure 4:**
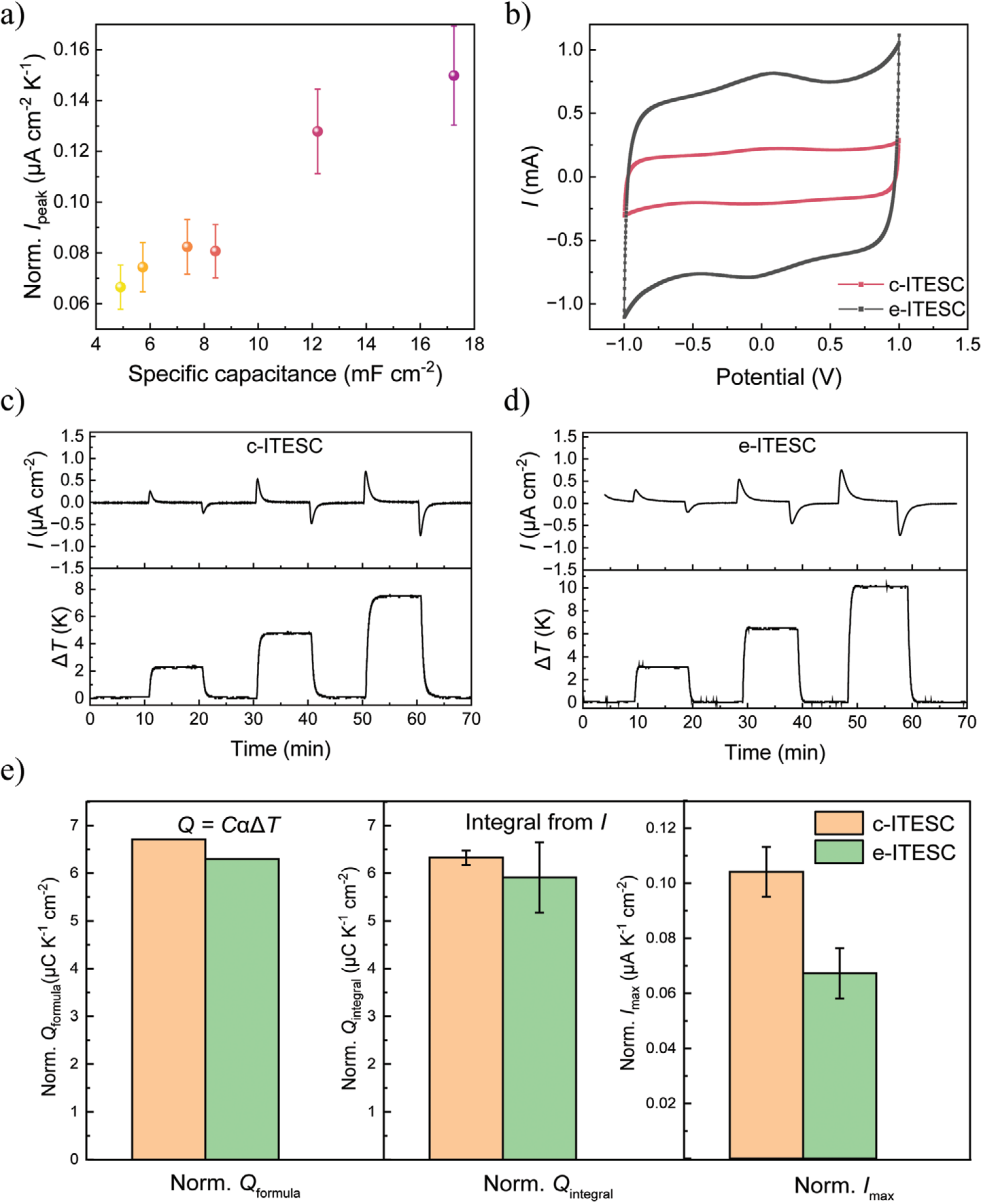
Effects of capacitance on resistive sensor operation. a) Normalized *I*_peak_ as a function of specific capacitances with error bars based on different ∆*T*. b) CV curves for ITESCs with circular (c‐ITESC, Φ = 1 cm, S = 0.79 cm^2^) or ellipse (e‐ITESC, 1 × 0.8 cm, S = 2.51 cm^2^) electrodes. c) Output current (top panel) and ∆*T* (bottom panel) of c‐ITESC d) Output current (top panel) and ∆*T* (bottom panel) of e‐ITESC. e) Amount of normalized transferred charge *Q_formula_*, *Q_integral,_* and *I*_peak_ during charging processes. The error bars are the standard deviation from three repeated cycles.

After validating the correlation of the external current with d∆*T*/dt, we investigate the impact of capacitance in this section. ITESCs with different capacitance (same series of sample as in Figure 2) were connected with an external load of 1 kΩ and the output voltage and current were recorded (Figure S5, Supporting Information). As shown in **Figure** **4**a, *I*_peak_ of the ITESCs increases with the specific capacitances. However, the *I*_peak_ only increases by 1.3 folds for an increase in capacitance of 2.5 folds (comparison between the devices of smallest and largest capacitance). This could be due to the decrease in Seebeck coefficient for ITESCs with larger capacitance. Moreover, for Equation. 1 we consider the development of thermal voltage and charging of ITESCs to take place immediately with the establishment of Δ*T*. However, the zoom‐in curve (Figure 3b) already indicated that there is a delay in those processes. The time needed for ionic thermodiffusion can be considered constant for the same electrolyte and electrode separation. The time constant τ for larger capacitance increases (τ  =  *RC*  = (*R_i_* + *R_load_*) *C*), *R*_i_ is the ionic resistance in the electrolyte and *R*_load_ is the load resistance). The *R*_i_ of the studied ITESC is in the range of 21.4 –42.5 Ω (Figure S6, Supporting Information), the change of which can be neglected compared to the 1000 Ω of the load resistance connected here. Hence, the time constant mainly scales with the capacitance, which could lead to a more extended current but less increase in the peak value.

In order to further understand the influence of the capacitance on *I*_peak_, we compared two devices with the same capacitance per area but different electrode areas (*S*) and therefore different total capacitances. This could reduce the impact of decreasing Seebeck coefficient when using electrodes with large capacitance. Two electrodes of different area (circular (radius 0.5 cm, *S* = 0.79 cm^2^) and ellipse (radii 1 and 0.8 cm, *S* = 2.51 cm^2^)) were prepared with similar CNTs loading per unit area (1 mg for circular and 3.2 mg for ellipse). The extracted capacitance from CV curves in Figure 4b is 9.6 mF for the circular ITESC (c‐ITESC) and 35 mF for the ellipse ITESC (e‐ITESC). α extracted from the linear fitting slope of ∆*V* versus ∆*T* shows similar values of 0.55 and 0.45 mV K^−1^ for the two devices (Figure S7, Supporting Information). The small difference is likely due to the differences between the CNTs loading per area (12.2 mF cm^−2^ for circular ITESC (c‐ITESC) and 14.0 mF cm^−2^ for ellipse ITESC (e‐ITESC)) and it is negligible compared to the more than 3 times larger difference in capacitance between the two devices. Figure 4c,d show the current curves of the c‐ITESC and e‐ITESC when applying similar ∆*T*. We observed that the e‐ITESC took longer time to charge and discharge compared to c‐ITESC. This is likely due to the capacitance of the e‐ITESC being 3 times larger compared to that of the c‐ITESC, while the total resistance is still determined by the relatively large load (Figure S8, Supporting Information). Figure 4e presents a comparison between the c‐ITESC and the e‐ITESC in terms of amount of *Q*_formula_ (determined by *Q_formula_* =  *CV*  =  *C*αΔ*T*), *Q*_integral_ (integrated current from charging), and normalized *I*_peak_ (normalized by area and Δ*T*). The normalized transferred charge per area (both from calculation and measurement) is similar for the two devices as expected. However, the e‐ITESC with larger capacitance shows lower *I*_peak_ per area compared to the c‐ITESC after normalization with 𝑑Δ𝑇/𝑑𝑡. This corroborates our hypothesis that a large capacitance increases the time constant of the supercapacitor and limits the normalized *I*_peak_. We plotted the accumulated *Q* over time for the c‐ITESC and e‐ITESC and marked the needed time for reaching 0.63 *Q* (0.8 min for c‐ITESC and 2.4 min for e‐ITESC) in Figure S9 (Supporting Information). The evident difference between c‐ITESC and e‐ITESC can be ascribed to the time constant τ that scales with the capacitance. The results show that increasing the capacitance could improve the peak current generated from ITESC. The enhancing effect will be reduced by the decreasing Seebeck coefficient and extended time constant for charging and discharging the capacitor. In addition, the time needed to fully discharge the ITESC should be considered for practical applications.

### 2.5 ITESCs with Resistive Sensor

**Figure 5:**
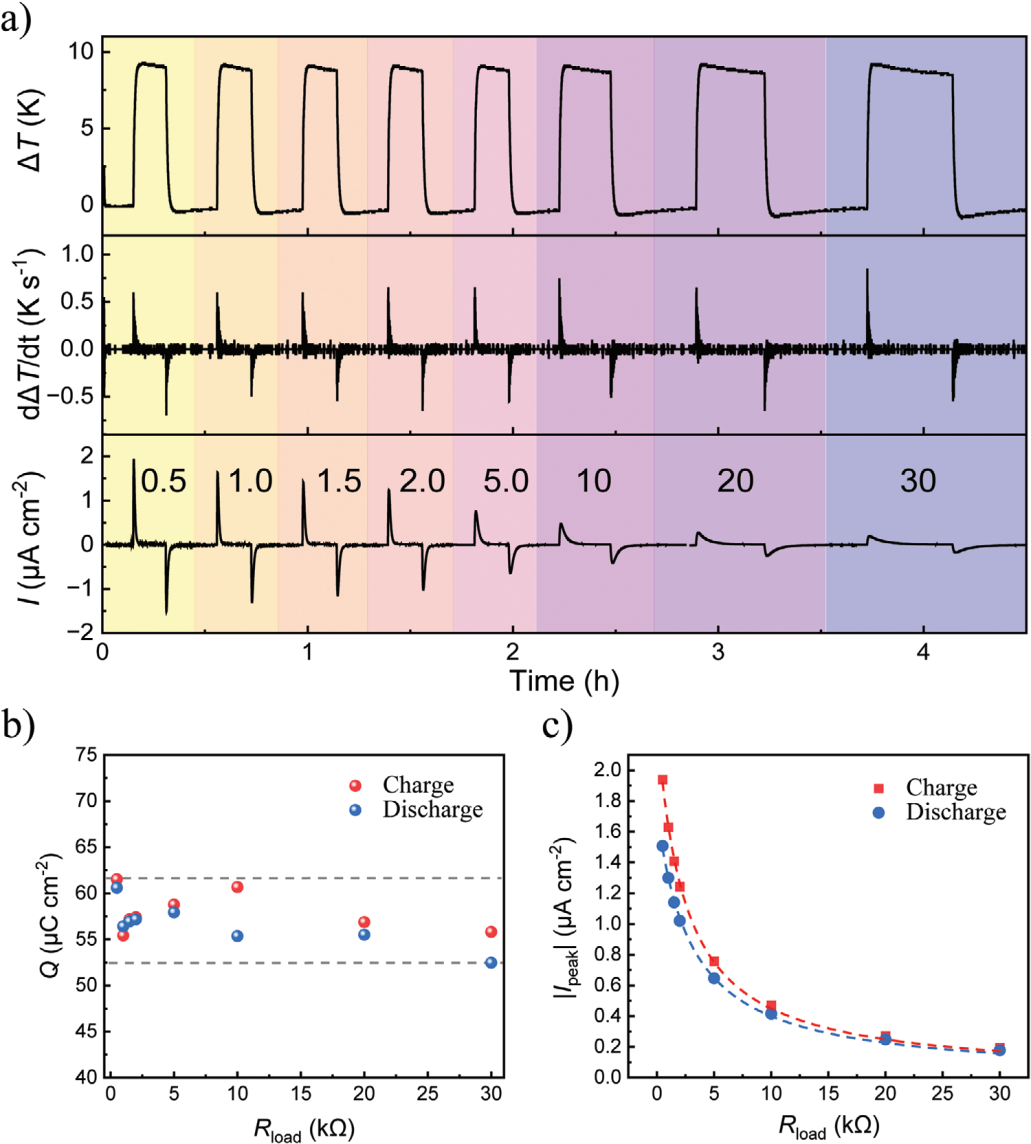
Connecting ITESC to different load resistance. a) Applied ∆*T* (top panel), d∆*T*/dt (middle panel) and output current (bottom panel) of an ITESC with different loads (0.5–30 kΩ). b) Amount of transferred charge *Q* for charging and discharging together for different loads. c) The absolute values |*I*_peak_| for ITESCs with different electrode loads upon charging and discharging process. The dashed curves are reciprocal fit to the experimental results.

After understanding the key impact of Δ*T* rate change and capacitance on the external current of an ITESC, we are ready to demonstrate the dependence of *I*_peak_ on the external load resistance. We connected a range of resistances (from 0.5 to 30 kΩ) to an ITESC with 12.2 mF capacitance, and applied ∆*T* of 9 K (top panel in **Figure** **5**a). As shown in the bottom panel in Figure 5a, the corresponding output current decreased with increasing load resistance for approximately the same 𝑑Δ𝑇/𝑑𝑡 (middle panel). It is further clear that the time needed to charge the ITESC shows an increasing trend with larger load resistances, due to the increase of time constant τ. By integrating the current over time, we obtained the amount of transferred charge from charging and discharging cycles. As shown in Figure 5b, the amount of charge for different loads did not vary significantly and stayed in the range between 53–62 µC cm^−2^. However, we noted a small decrease in the transferred charge with increasing the resistance of the load, which could be due to increased time constant of the capacitor and/or increased Joule heating that consumes energy and reduces charging efficiency. Figure 5c presents the *I*_peak_ from charge and discharge curves for different load resistances, showing a clear inversely proportional relationship. This is expected from the correlation $I_{peak}\propto \frac{V_{thermal}}{R_{load}+R_{i}}$, where *V*_thermal_ is the open circuit voltage driven by the applied ∆*T*. The internal resistance R_i_ (22 Ω, Figure S6, Supporting Information) is relatively small compared to the connected load from 500 to 30 000Ω.

**Figure 6:**
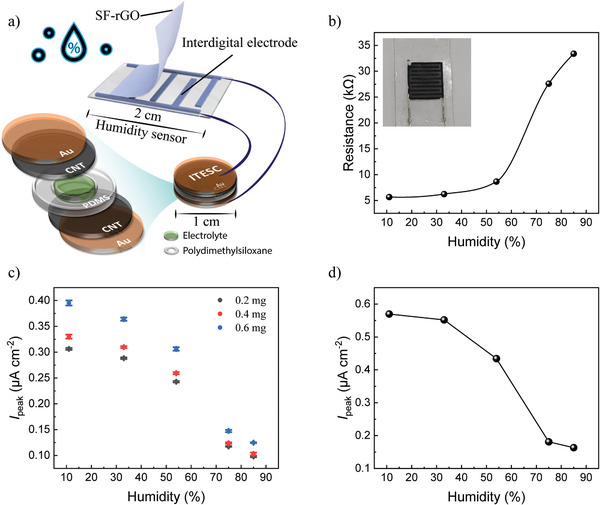
Demonstration of resistive sensor powered by ITESC. a) Schematic of an ITESC integrated with humidity sensor. b) The resistance of a humidity sensor as a function of humidity. c) *I*_peak_ for ITESCs with electrodes loaded with 0.2, 0.4 and 0.6 mg CNTs as a function of humidity. The error bars are the standard deviation from three repeated cycles under the same heating conditions. d) The *I*_peak_ for ITESC with electrodes loaded with 1 mg CNTs under the same heating conditions as a function of relative humidity.

To determine the performance of operating a resistive sensor by the charging current from ITESC, we connected a humidity sensor as the load (as depicted in **Figure** **6**a). The humidity sensor consisted of silk fibroin and reduced graphene oxide composites (SF‐rGO) as active sensing layer, which was deposited on top of screen‐printed interdigital electrodes. When water (electron donor) adsorbs onto the rGO (p‐type semiconductor) film, the hole concentration of the rGO decreases and potential barriers for the carriers form, resulting in an increase in resistance.^[^ ^28^ ^]^ Meanwhile, water molecules also swell the SF, leading to an increase in the distance between rGO and SF sheets.^[^ ^29^ ^]^ The special structural characteristic of SF (tunable secondary structure) and rGO (honeycomb‐like nanosheet) of the prepared sensor is the key to high sensitivity toward humidity. SEM images and Raman spectra of GO and SF‐rGO are shown in Figure S10 (Supporting Information). To evaluate the sensitivity and the resistance range of the sensor, we first characterized the corresponding resistance of the sensor exposed to different relative humidity (RH). As shown in Figure 6b, the resistance increases from 5 to 35 kΩ for RH changing from 10% to 80%. Moreover, the resistance of the humidity sensor shows a quasi‐exponential response as the humidity rises, with a rapid increase in resistance occurring ≈55% humidity. Therefore, we can achieve a higher resolution of humidity above 55% for this specific humidity sensor when there is no limitation on the minimum current reading. Interestingly, this feature compensates for the smaller dependence of *I*_peak_ on resistance for larger resistances (Figure 5c).

To balance the trade‐off between response range and response time, we evaluated the performance of the sensor driven by ITESCs of three different capacitances (electrodes loaded with 0.2, 0.4, and 0.6 mg of CNTs, respectively). As shown in Figure 6c, the variation of *I*_peak_ is generally larger for ITESCs with large capacitance, and the response for humidity below 50% is not obvious for ITESCs with small capacitance (original curves shown in Figure S11, Supporting Information). Considering the improved sensitivity and increasing response time for large capacitance, we chose ITESC with an electrode loaded with 1 mg to characterize sensor performance for the whole humidity range. As shown in Figure 6d, we observed noticeable changes in *I*_peak_ from 11% to 85% humidity. The result proves the feasibility of directly using the charging and discharging current from ITESC to operate resistive sensors for real‐time measurements.

## 3 Conclusion

In summary, we have demonstrated the direct use of the charging/discharging current from ITESCs to operate resistive sensors. The peak current from charging and discharging an ITESC could be correlated to the resistance of a connected sensor, thereby providing information of changes of targeting signals. For practical applications, we further demonstrate how the effect of temperature variation on the current and sensor response could be compensated by measuring the output voltage and current synchronously. We provide proof of concept measurements by using ITESCs to power a resistive humidity sensor, showing clear correlation between humidity and the generated current signals. This concept directly utilizes the ionic thermoelectric effect with minimal energy loss, offering a new way to power portable sensors.

## 4 Experimental Section

### Vertical ITESC Fabrication

CNTs (0.1 g, multiwall, carboxylic acid functionalized, Sigma–Aldrich) were dispersed in 30 mL of deionized water using a homogenizer (IKA ULTRA‐TURRAX) for 15 min followed by ultrasonic treatment for 3 h. The dispersed mixtures with a series of volumes were drop casted onto glass substrates with thermally evaporated Au surfaces (Φ 1 cm for c‐ITESC, or 1 × 0.8 cm for e‐ITESC, thickness of 70 nm) to achieve electrodes with varying amounts of CNTs deposition. The electrolyte were used in this study was a gel‐like electrolyte combining the ionic liquid 1‐ethyl‐3‐methylimidazolium ethyl sulfate (EMIM:ES) with 10% hydroxyethyl cellulose (HEC). Cured polydimethylsiloxane (PDMS) pieces, featuring a cut‐out chamber for the iTE electrolyte, were employed to separate the Au electrodes at 2 mm distance. The ITESC was sealed using uncured PDMS and cured at 70 °C, creating a thin separation layer to prevent any effect from the outside.

### Lateral ITESC Fabrication

The Au electrodes loaded with CNTs were fabricated in the same way as vertical ITESC. The two electrodes were placed on the horizontal plane substrate and connected with an electrolyte applied in between. The lateral ITESC is only used in Figure S3 (Supporting Information) to investigate the effect of applied temperature difference rate.

### Humidity Sensor Fabrication

The humidity sensor was fabricated in three steps. a) Preparation of (silk fibroin) SF solution: 8 g bombyx mori cocoons were first boiled in 500 mL NaHCO_3_ (0.02 m) solution for 30 min to remove the sericin.^[^ ^8^ ^]^ After washing by distilled water, the obtained silk fibers were dried at 60 °C overnight. 3 g of the dried samples were immersed in 100 mL of LiBr (9.3 m) solution and heated to 60 °C for 4 h, followed by dialysis against deionized water for 48 h to obtain SF solution. b) Preparation SF‐rGO solution: As‐prepared SF solution was added in 100 mL graphene oxide (GO, 50 mg) solution under stirring at a mass ratio of SF/GO = 1:1. The pH value of the mixture was adjusted to 10 using NH_4_OH. 1.60 g glucose was then added into the mixture as a reductant, and the solution was stirred for 5 h at 95 °C. After washing, freeze‐drying, and redissolving, SF‐rGO composites were successfully synthesized. c) Fabrication of the humidity Sensor: The interdigital electrodes were designed by AutoCAD software. And then commercial silver ink was used for the fabrication of the electrode via screen‐printing technology. After being cured at 60 °C for 30 min, the electrode was modified with 600 µL SF‐rGO solution. The sensor array was finally obtained by being dried in an oven at 60 °C for 3 h.

### Thermal and Electrochemical Experiments

All temperatures were measured by thermocouples (K type, Pentronic AB) with a digital thermometer (TMD‐56, AMPROBE). The applied temperatures were controlled by Peltier element (5×5 cm, Adaptive) with silicon pad on the surface powed by series source meter (Keithley 2400). A nanovoltmeter (2182A, Keithley) was used to monitor open circuit voltages and voltages across the resistor. Impedance spectra were acquired using an impedance spectrometer (Alpha High Resolution Dielectric Analyzer, Novocontrol) in the frequency range from 10–2 to 107 Hz with the amplitude of 5 mV. The capacitance was calculated by: *C*  = −1/(2π*fZ*′′) , where *f* was the frequency and *Z*′′ was impedance of imaginary axis. Cyclic voltammetry (CV) measurements were carried out with an electrochemical workstation (SP‐200 Pontentiostat, BioLogic) at a scan rate (ν) of 20 mV s‐1. The capacitance was calculated by: $C=\frac{1}{2\nu (V_{2}-V_{1})}\;\int_{V_{1}}^{V_{2}}idV$, where *V*_1_ and *V*_2_ represent the scanned potential range.

## Conflict of Interest

The authors declare no conflict of interest.

## Supporting information



Supporting Information

## Data Availability

The data that support the findings of this study are available from the corresponding author upon reasonable request.
