# Supplementary material for: Ionic Thermoelectric‐Powered Resistive Sensors
Source: Adv Sci (Weinh). 2024 Dec 16;12(6):2413093. doi: 10.1002/advs.202413093 (PMC11809375; doi:10.1002/advs.202413093)
Supplement: Supplementary file 1 — Supporting Information [file ADVS-12-2413093-s001.docx]

**Supplementary Information for**

**Ionic Thermoelectric-Powered Resistive Sensors**

Mingna Liao^1,2^, Hongting Ma^3^, Nan Zhu^3^*, Magnus Jonsson^1,2^ and Dan Zhao^1,2^*

*^1^Laboratory of Organic Electronics, Department of Science and Technology, Linköping University, Norrköping SE-601 74, Sweden.*

*^2^Wallenberg Wood Science Center, Linköping University, Norrköping SE-601 74, Sweden.*

*^3^School of Chemistry, Dalian University of Technology, Dalian, Liaoning 116024, China.*


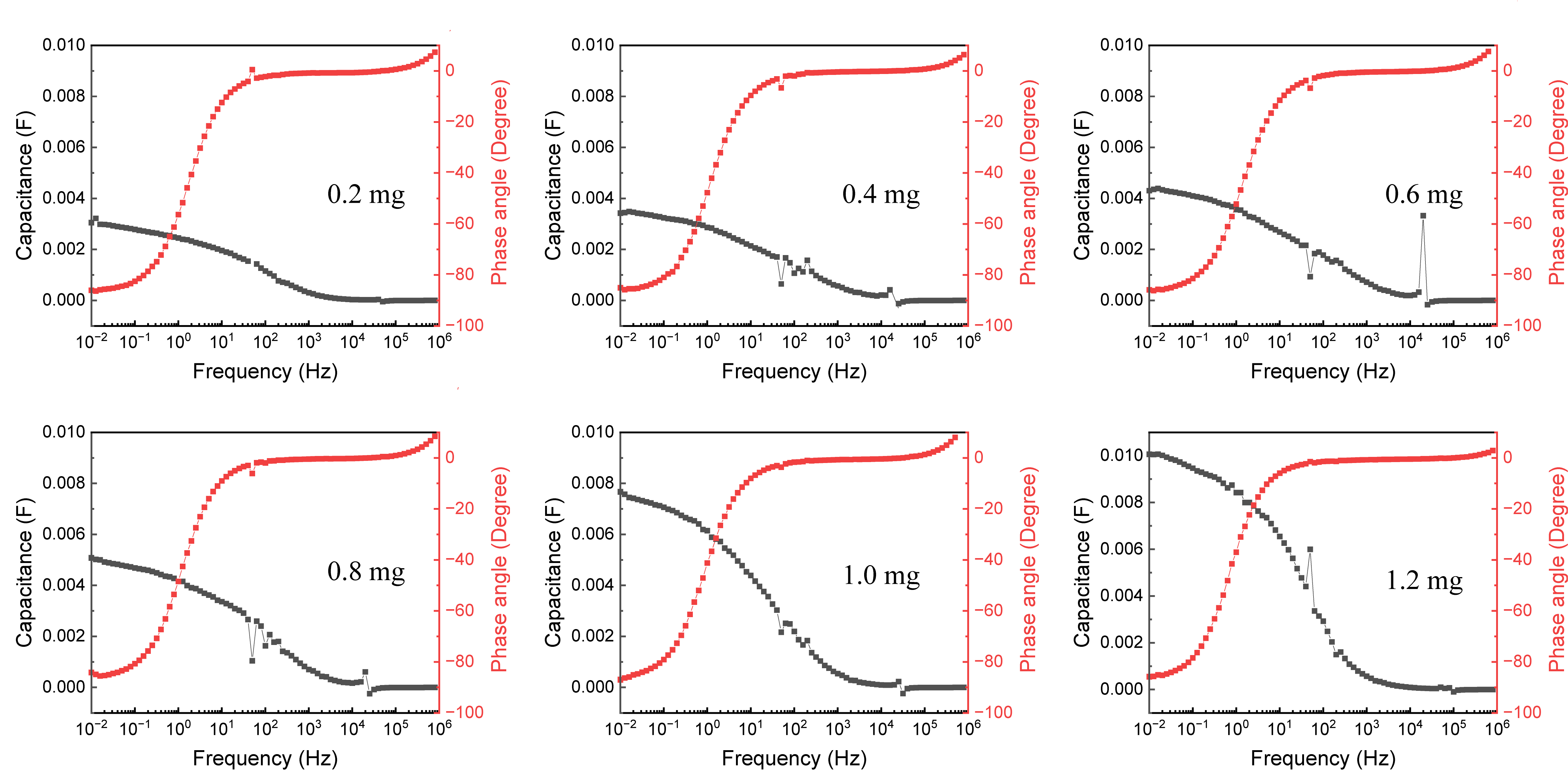


**Figure S1.** Phase angle and capacitance calculated from impedance data by formula $C={-1}/{(2\pi fZ^{''})}$, from results for ITESCs with different CNTs loads on their electrodes (indicated directly in the panels).

**Figure S2.** The peak values of d∆*T*/dt for different ∆*T* with linear fitting.

**
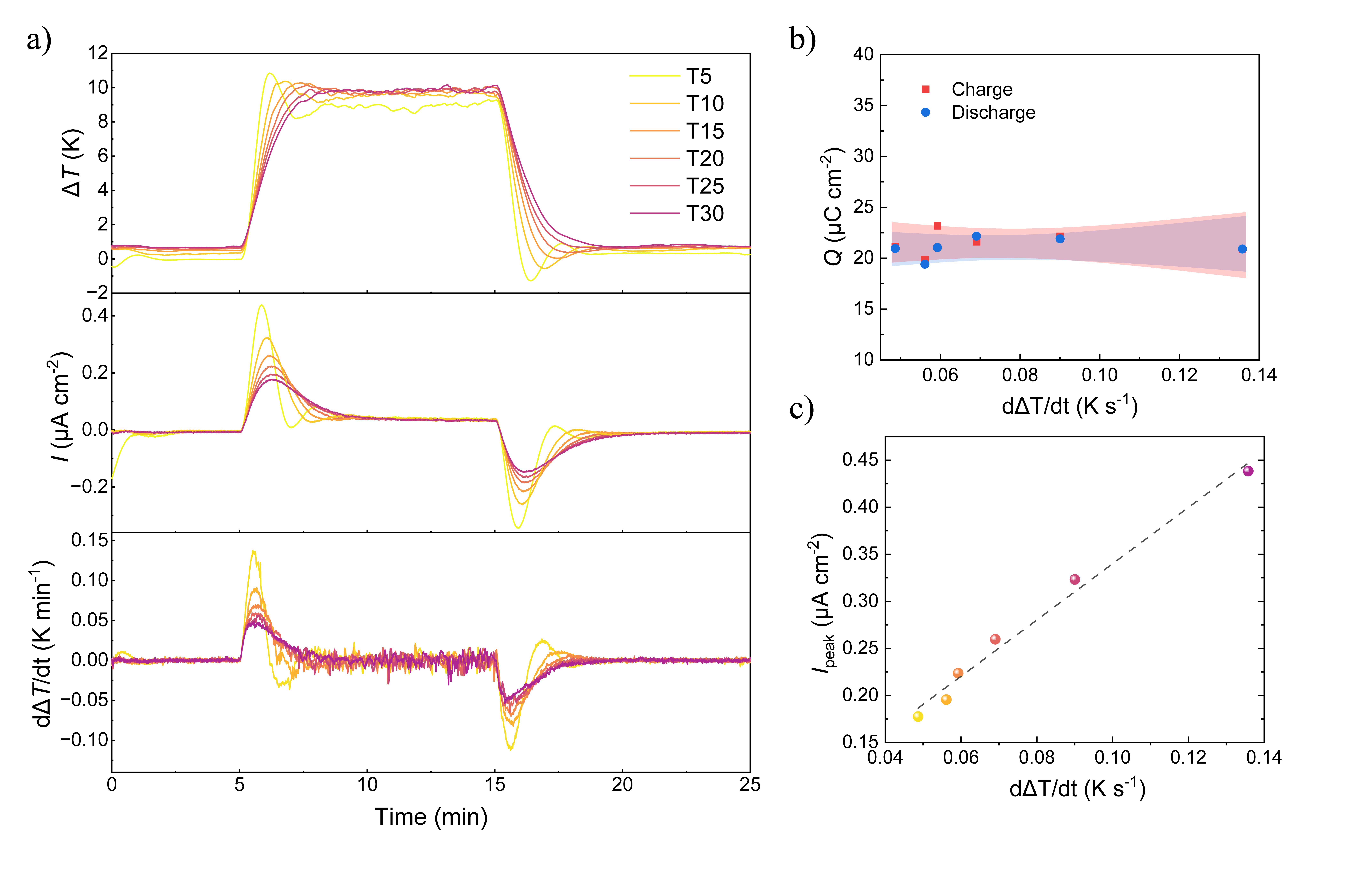
**

**Figure S3 | The effect of applied temperature difference rate. a**) Output current (top panel), ∆*T* (middle panel) and d∆*T*/dt (bottom panel) of an ITESC for different rates (T5-T30). **b**) Amount of transferred charge for charging and discharging together with 95% confidence range (charging in red and discharging in blue). **c**) The *I*_peak_ of charging processes for different d∆*T*/dt.

**Note S1**: In order to confirm the direct correlation between the *I*_peak_ and 𝑑Δ𝑇/𝑑𝑡, we applied/removed the same ∆*T* (10 K) at different rates (T5 to T30). The output current curves (top panel), ∆*T* (middle panel) and 𝑑Δ𝑇/𝑑𝑡 (bottom panel) were plotted in Figure S3a. The amount of transferred charge during charging and discharging were calculated by integrating the current and shown in Figure S3b. It can be observed that the confidence ranges for the charging and discharging charges are mostly within the range of 20-23 µC cm^-2^, regardless of the ∆*T* rates. This confirms that the amount of transferred charges are solely determined by the applied ∆*T* with the constant ITESC capacitance. The *I*_peak_ of charging processes increases linearly with the d∆*T*/dt (Figure S3c).


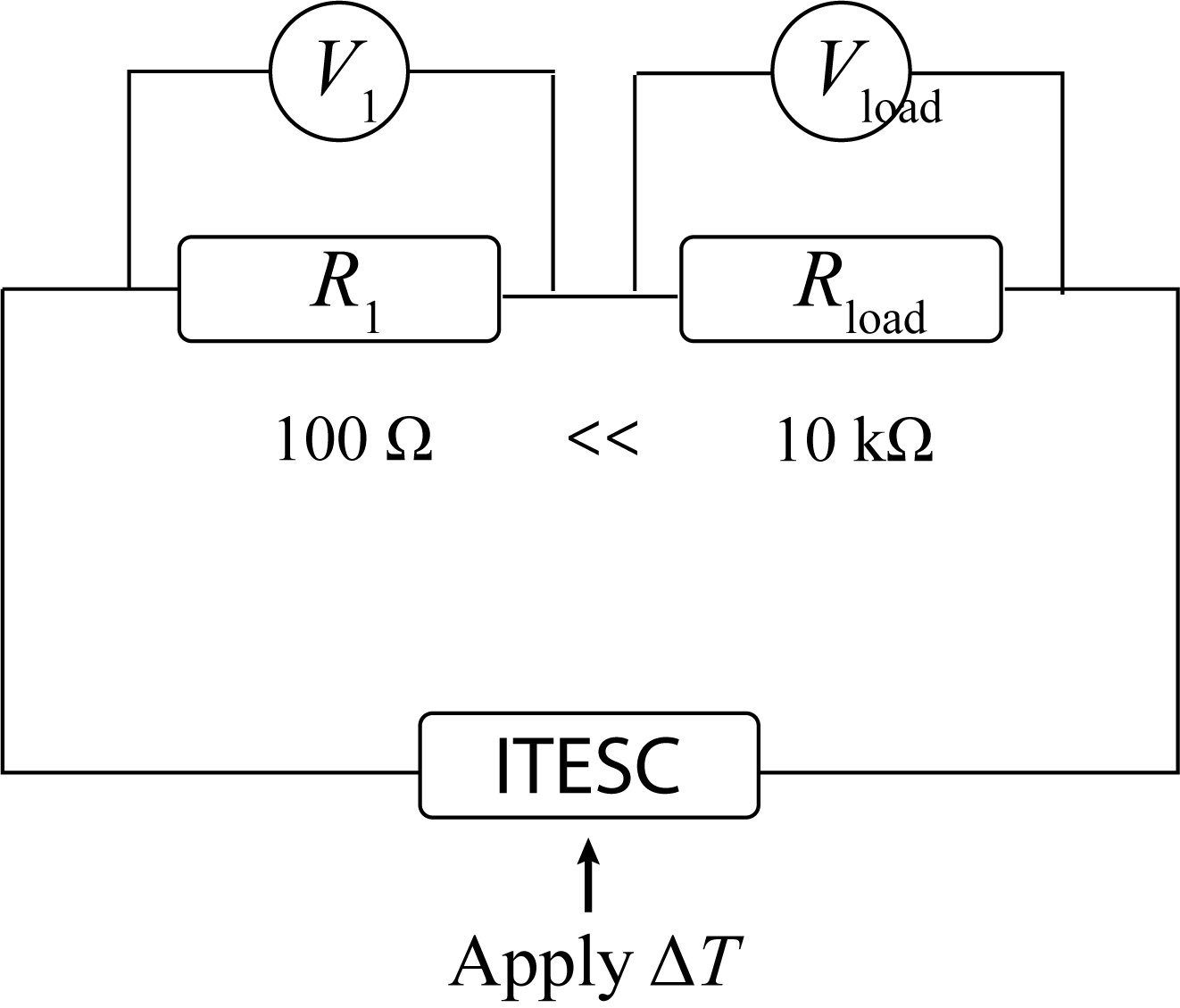


**Figure S4.** Schematic of the experimental circuit.


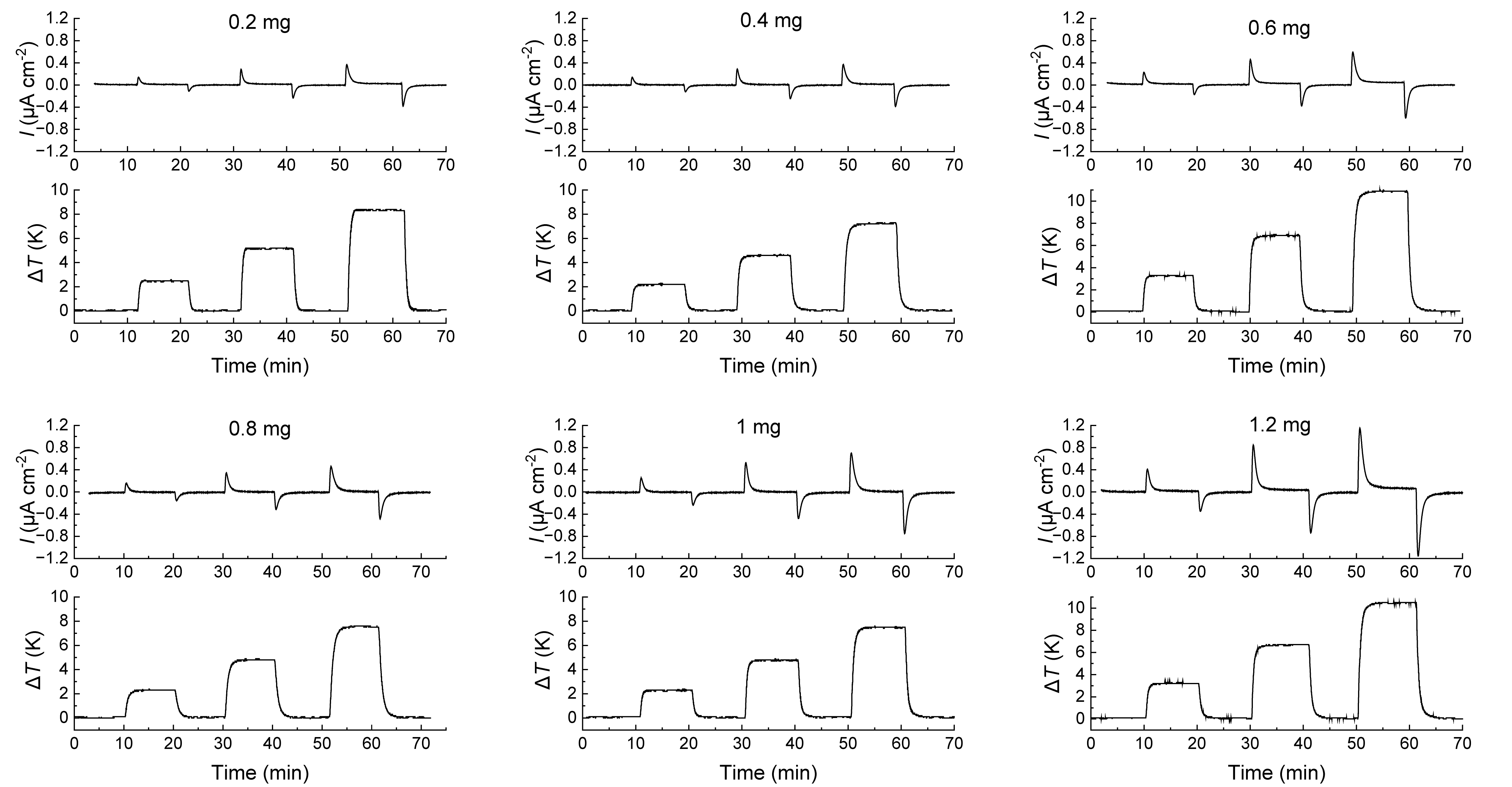


**Figure S5.** Output current under increasing ΔT across ITESCs with different CNTs loading.

**Figure S6.** Impedance of real axis $Z^{'}$ as a function of phase angle for ITESC with different CNTs loading.

**Figure S7.** Open circuit voltage changes (∆*V*) as a function of ∆*T* across the c-ITESC and e-ITESC.

**
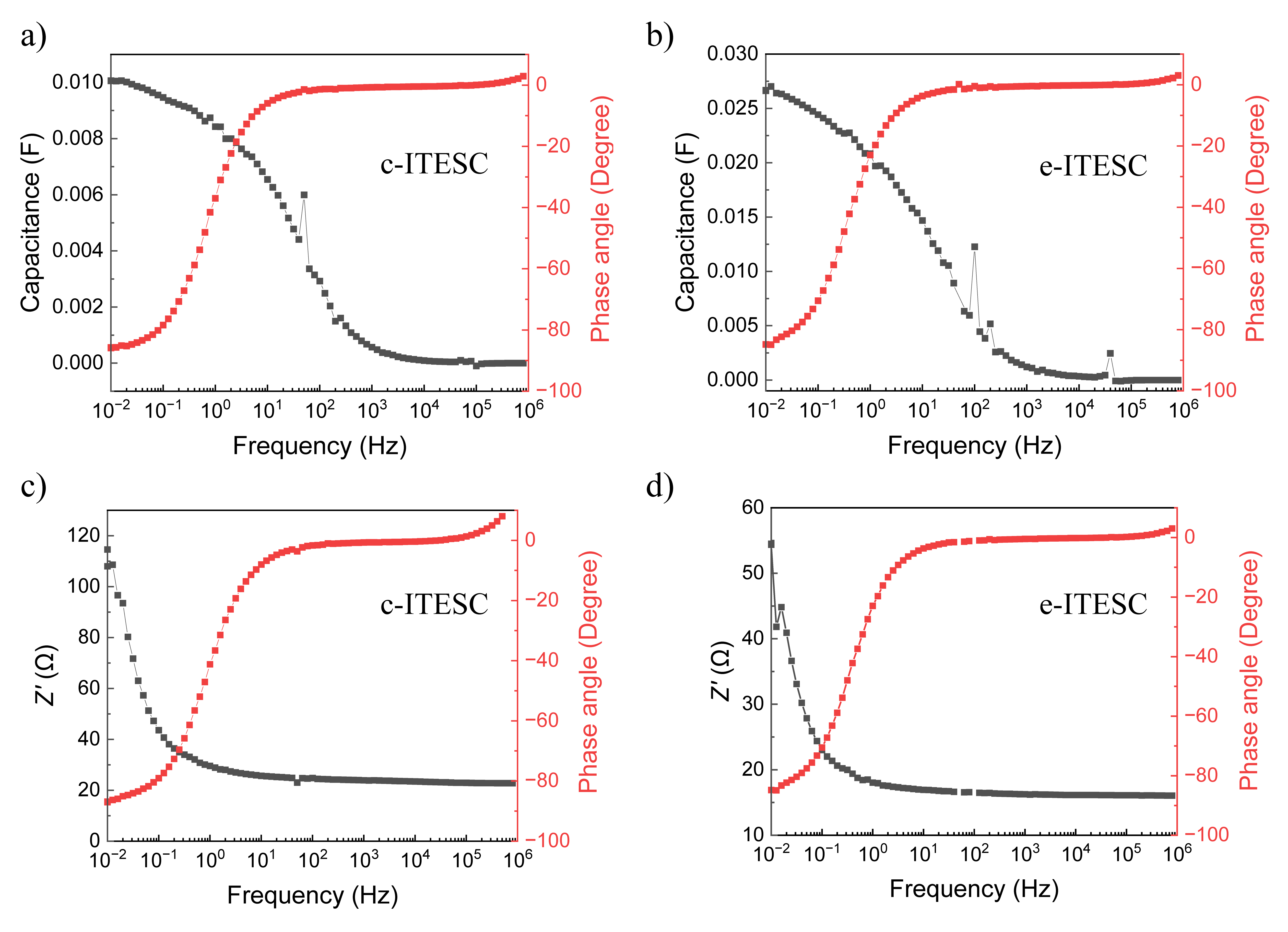
**

**Figure S8.** Phase angle and capacitance calculated from impedance data by formula $C={-1}/{(2\pi fZ^{''})}$, from results for c-ITESC (**a**) and e-ITESC (**b**). Phase angle and resistance $Z'$ from impedance data for c-ITESC (**c**) and e-ITESC (**d**) (indicated directly in the panels).


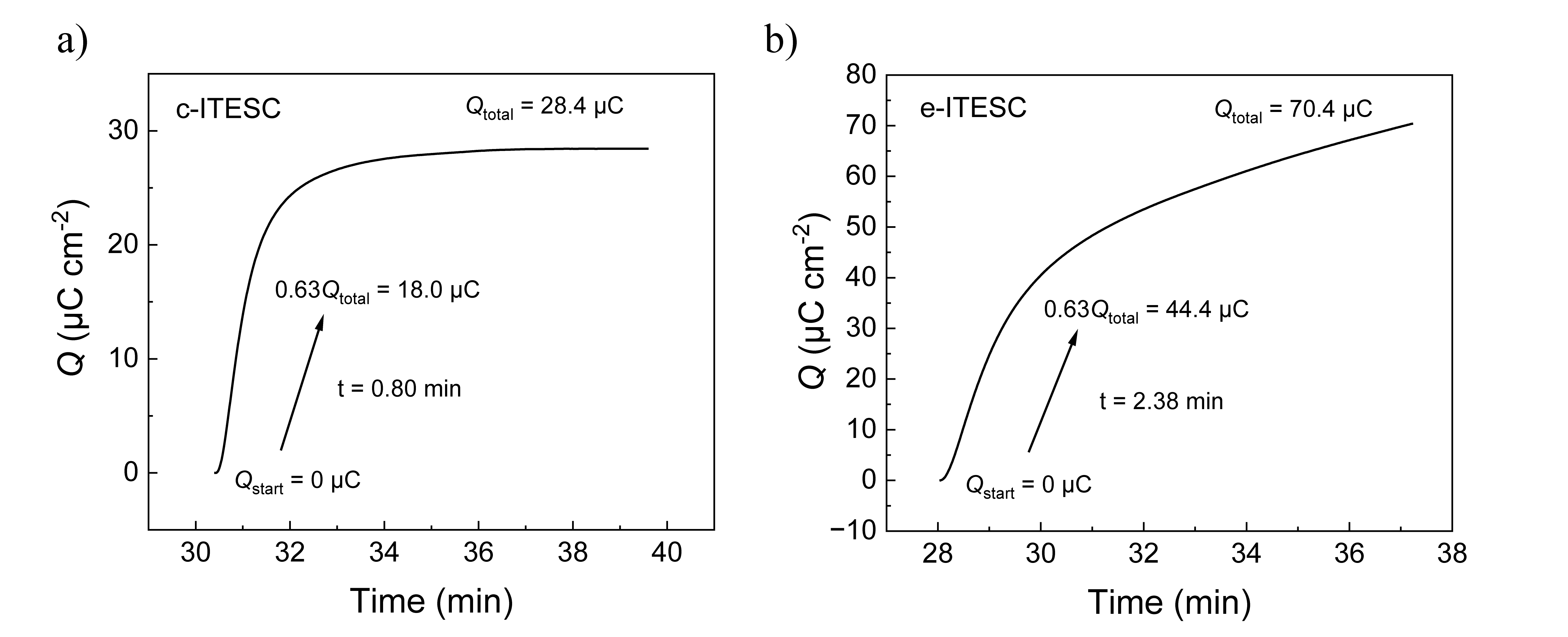


**Figure S9.** Amount of transferred charge *Q* for c-ITESC (**a**) and e-ITESC (**b**) and the needed time for reaching 0.63 *Q* (0.8 min for c-ITESC and 2.4 min for e-ITESC).

**Note S2:**

SEM images show the architecture of GO and SF-rGO (Figure S10a-b). The surface of the GO and SF-rGO composite are both characterized as continuous films with abundant wrinkled structures. Meanwhile, agglomerated structure of SF was observed in SF-rGO, indicating the successful loading of SF into rGO. Raman spectra revealing the functional group, defects and bonding characteristics for GO and SF-rGO are shown in Figure S10c. D-band occurs at 1352 cm^-1^ and G band appears at 1601 cm^-1^ in GO. D-band arises from breathing modes of six-atom ring activated by structural defects/disorder or interruption in the symmetric sp^2^ carbon bonds, while G-band is due to the in-plane lattice vibration of sp^2^ carbon atoms. The intensity ratio (*I*_D_/*I*_G_) is a measure for the degree of defects in graphene materials. *I*_D_/*I*_G_ decreased from 0.85 to 0.83 in SF-rGO, indicating oxygen removal, as compared to GO.


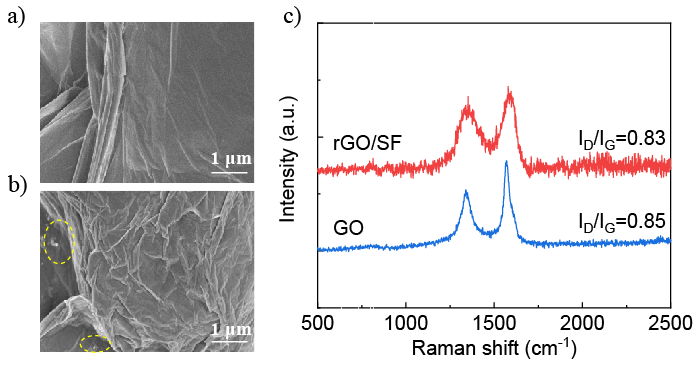


**Figure S10.** SEM images of a) GO and b) SF-rGO. b) Raman spectra of GO and SF-rGO.

**
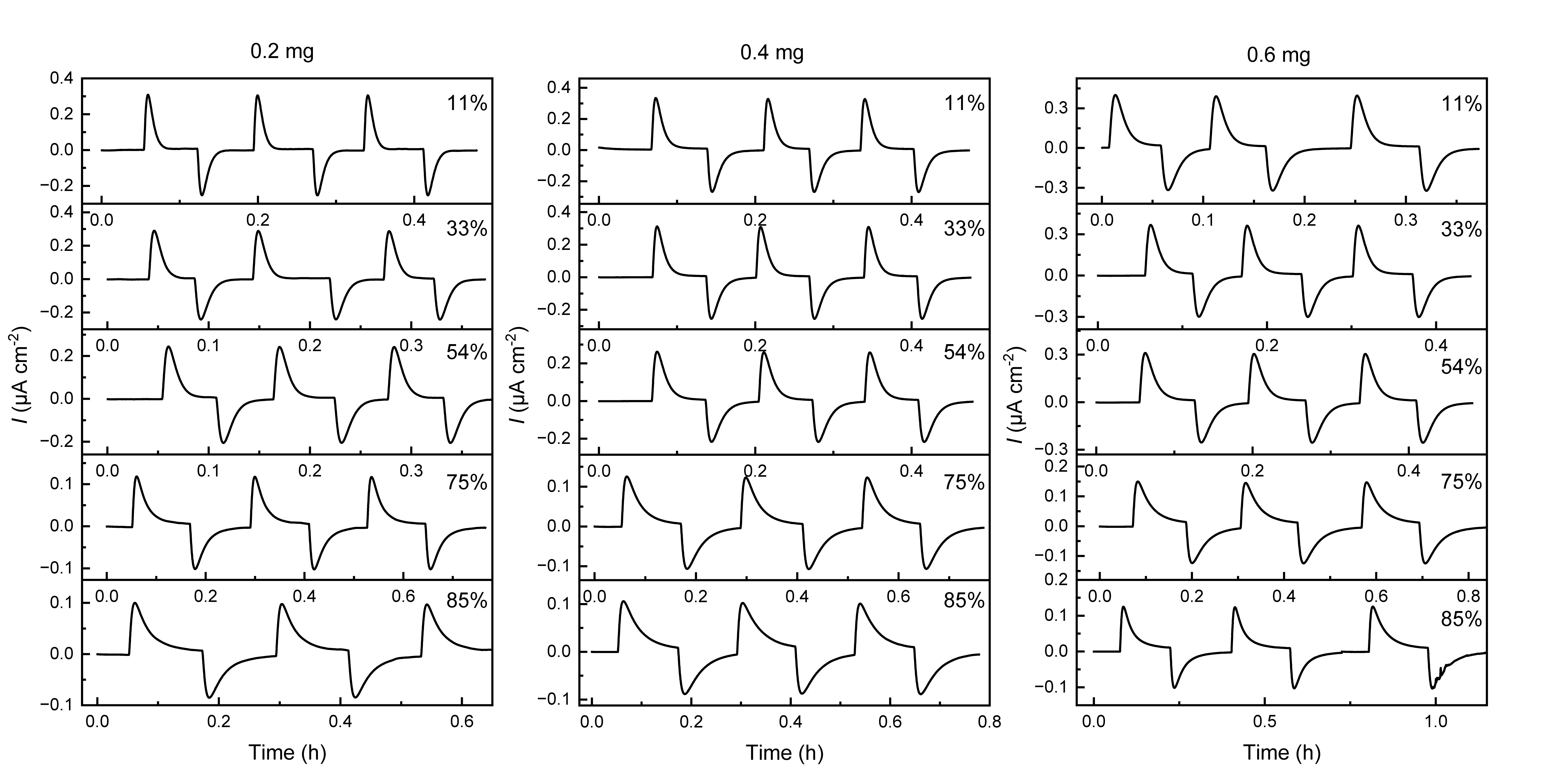
**

**Figure S11.** Output currents measured at 11%, 33%, 54%, 75% and 85% humidity for ITESCs with 0.2, 0.4 and 0.6mg CNTs loading, respectively.
